# Coccidia Vaccine Challenge and Exogenous Enzyme Supplementation in Broiler Chicken 2—Effect on Apparent Ileal Nutrient and Energy Digestibility and Intestinal Morphology 7 and 14 Days Post-Challenge

**DOI:** 10.3390/ani15030401

**Published:** 2025-01-31

**Authors:** Sunday A. Adedokun, Andrew Dunaway, Richard Adefioye

**Affiliations:** Department of Animal and Food Sciences, University of Kentucky, Lexington, KY 40546, USA

**Keywords:** broiler chicken, challenge, corn, enzyme supplementation, performance, wheat

## Abstract

The effect of exogenous mixed-enzyme supplementation (xylanase, β-glucanase, and pectinase) and coccidia vaccine challenge (CVC, Coccivac B-52™) on broilers fed a corn–SBM (CS) and a wheat–CS (WCS)-based diet was examined in this study. On day 14, 448 Cobb by-product breeder male broiler chickens were assigned to treatments (factorial arrangement) in a completely randomized design, with each treatment replicated seven times. Treatment effect was evaluated within each diet type as a 2 (enzyme levels) x 2 (CVC, 0 or 20X) factorial arrangement of treatments 7 and 14 days post-CVC. The 7-day (days 14–21) post-CVC, BWG, and feed efficiency (birds on the CS-based diet) were lower (*p* < 0.05), while birds on enzyme-supplemented diets had higher (*p* < 0.05) BWG compared to birds on diets without enzyme supplementation. Between days 21 and 28, an interaction between CVC and exogenous enzyme resulted in higher (*p* < 0.05) BWG compared with the challenged birds fed diets without enzyme supplementation. For birds fed WCS-based diets, CVC influenced (*p* < 0.05) BWG and feed efficiency (decreased days 14–21 and increased days 21–28), while CVC birds had higher BWG and feed efficiency 14 days post-CVC. Apparent ileal digestibility of dry matter, energy, and DE were lower (*p* < 0.05) in CVC broilers fed either the CS- or WCS-based diets (7 and 14 days post-CVC). Interaction between CVC and exogenous enzyme supplementation indicated that CVC, irrespective of exogenous enzyme supplementation with the WCS-based diet, decreased (*p* < 0.05) Ca utilization (7 days post-CVC) but increased (*p* < 0.05) Ca utilization compared to CVC birds without enzyme supplementation 14 days post-challenge. Seven days post-CVC, irrespective of the diet type, CVC resulted in lower (*p* < 0.05) duodenal VH and VH:CD and higher (*p* < 0.05) CD. Enzyme supplementation influenced (*p* < 0.05) duodenal CD (increased) and VH:CD (decreased) in birds fed the WCS-based diet. Results from this study showed that complete recovery from CVC was influenced by diet type, with CVC birds fed WCS-based diet having higher BWG and feed efficiency compared to the unchallenged birds.

## Simple Summary

The efficiency with which the nutrients and energy in any diet are digested, absorbed, and utilized depends on several factors, with the health status of the bird’s gastrointestinal tract playing an important role. The protozoan parasite *Eimeria* has been reported to significantly reduce the performance of birds because of poor absorption of nutrients in the small intestine. In this study, the effect of *Eimeria* sp. challenge and exogenous enzyme supplementation on performance and nutrient digestibility in broilers fed corn-soybean meal- or wheat-corn–SBM-based diets was examined. During the first 7 days post-challenge (days 14–21), in birds fed the corn–soybean-meal-based diets, the coccidia vaccine challenge (CVC) resulted in lower BWG and feed efficiency, while birds on the enzyme-supplemented diets had higher BWG compared to birds on diets without enzyme supplementation. However, the CVC birds fed the wheat–corn–soybean-meal-based diets performed better (BWG and feed efficiency) compared to the unchallenged birds during the recovery phase (days 21–28). Findings from this study showed that the effect of CVC on broiler chickens’ performance during the recovery phase may be influenced by diet type.

## 1. Introduction

The gastrointestinal tract (GIT) of poultry plays important roles in the storage and movement of feed and digesta as well as in the digestion and absorption of nutrients. By virtue of these functions, the potential for exposure to biotic challenge is high. These challenges could be from bacteria, protozoa, or viruses. One of the most common forms of GIT challenges in broiler chickens is coccidiosis caused by the protozoa *Eimeria*, which has been reported to cause billions of dollars in annual economic loss to the poultry industry [1,2]. These losses arise from mortality as well as morbidities arising from the bird’s inability to efficiently digest, absorb, and utilize energy and nutrients from their feed [2,3].

Plant-based feed ingredients, such as soybean meal, canola meal, corn, wheat, barley, and rye, contain variable levels of soluble non-starch polysaccharides (NSPs), which may reduce the bird’s performance as a result of an increase in digesta viscosity. The negative effect of high digesta viscosity on birds’ performance could be attributed to the potential reduction in the ability of the endogenously secreted digestive enzymes to break down macromolecules because of the reduction in the available surface area in the GIT [4,5,6,7,8]. The levels and extent of this effect and the functionality of exogenous enzyme supplementation depend on the composition of the specific feed ingredients [9]. The two commonest energy-containing feed ingredients used in broiler diets are corn and wheat. Unlike corn, wheat has relatively higher levels of soluble NSP; hence, birds fed diets containing wheat without exogenous enzyme supplementation would be less efficient in utilizing the nutrients and energy in the diets. Different forms of exogenous enzymes, such as carbohydrases, are routinely supplemented to poultry diets to enhance energy and nutrient digestion [5,6,8,10]. These enzymes have resulted in enhanced performance of poultry in terms of energy and nutrient utilization by ameliorating the negative effect of the NSP in broiler diets. Exogenous carbohydrase enzyme supplementation of diets containing high levels of NSP ensures a reduction in digesta viscosity, which eventually reduces the amount of nutrients that eventually reach the hindgut, which could lead to a reduction in the incidence of Gram-negative bacterial [8,9,10].

The *Eimeria* challenge in poultry has been reported to decrease nutrient and energy digestion and absorption. The destructive effect of these parasites on the mucosa of the GIT coupled with a decrease in feed intake typically results in poor performance, increased morbidity, and, in severe cases, death [11,12]. In addition to this, *Eimeria* infection could result in the destruction of the intestinal barrier, which can open the door to opportunistic bacterial infections. Furthermore, due to its effects on nutrient digestion and absorption, the level of short-chain fatty acid producing bacteria and beneficial bacteria, such as *Bifidobacteriaceae* and *Lactobacillus,* could be reduced [13,14,15].

Presentation of a diet that is high in soluble NSP to birds that have been exposed to coccidiosis may further limit the bird’s ability to optimally digest, absorb, and utilize nutrients in the diet. However, the addition of certain carbohydrase enzymes to the diet may minimize these negative effects. In general, diets fed to broiler chickens are either corn- or wheat–SBM-based; it is therefore important to evaluate how birds on either corn- or wheat-based diets would respond to an *Eimeria* challenge. Furthermore, it is important to evaluate the relationship between these grains, the *Eimeria* challenge, and exogenous carbohydrase enzymes. Birds were raised in battery cages to avoid re-infection in order to quantify the effect of the different treatments on the selected response variables during the peak period and the recovery phase. Based on this, we examined the effect of CVC and exogenous enzyme supplementation to corn–soybean meal (CS)- and wheat–CS (WCS)-based diets on performance, nutrient and energy digestibility and utilization, and duodenal and jejunal histology in broiler chickens 7 (day 21; peak CVC) and 14 (day 28; recovery phase) days post-CVC.

## 2. Materials and Methods

The experimental procedures and birds’ management followed the standard operating procedures for the animal facility and were reviewed and approved by the University of Kentucky Animal Care and Use Committee (Protocol # 2018-2890) prior to the start of the study.

### 2.1. Birds, Diets, and Enzyme Supplementation

On day zero, 500-day-old male broiler by-product breeder chicks (Cobb) were individually tagged and fed a corn–SBM-based broiler starter diet containing 22, 0.9, and 0.45% of crude protein (N × 6.25), calcium (Ca), and non-phytate phosphorus, respectively. The study was conducted in battery cages in an environmentally controlled room with 20 h of light and 4 h of darkness. Birds had unrestricted access to feed and water throughout the duration of the study. On day 14, 448 birds (224 birds per diet type) were weighed and randomly assigned to treatments in each of the two studies in a completely randomized design [16]. Treatments were arranged as a 2 (challenge status) × 2 (supplemental enzyme status) factorial, resulting in four treatments per diet type, with seven replicates per treatment. There were eight birds per replicate cage. Four birds per cage (the two heaviest and two lightest birds) were sampled on day 21, while the remaining four birds per cage continued with the experiment until day 28, when they were sampled. Prior to sampling on days 21 and 28, birds were weighed individually and euthanized per cage using CO_2_ asphyxiation.

The control diet was the CS. The WCS was produced from the control diet by replacing 30% of the energy-yielding components (corn, SMB, and soy oil; Table 1) of the control diet with wheat. Similar ratios of the energy-yielding components of the diets were maintained across all of the experimental diets, as previously described [17]. The exogenous enzymes (Ronozyme^®^ WX2 and Ronozyme^®^ VP, DSM Animal Nutrition, Parsippany, NJ, USA) were supplemented to a portion of each of the CS- and WCS-based diets to produce the enzyme-containing diets. Ronozyme^®^ WX2 (xylanase) premix was added to the diet to supply 0.1 g/kg of the enzyme per kg of the diet, while Ronozyme^®^ VP (glucanase + pectinase) premix was added to the diet to supply 0.25 g of the enzyme per kg of the diet, as recommended by the manufacturer (DSM, Parsippany, NJ, USA). All diets, with or without enzyme supplementation, were produced from the same basal diet and were fed to the birds in mash form.

### 2.2. Coccidia Vaccine Challenge

To create the main factor of the challenge on day 14, half of the birds (the non-CVC treatments) were orally gavaged with 0.6 mL of distilled water, while the CVC birds were orally gavaged with 20X of coccidia vaccine (Coccivac^®^-B52, Merck Animal Health, Rahway, NJ, USA) diluted in 0.6 mL of distilled water, as previously described [17,18]. The 0.6 mL volume that was administered (gavaged) to 14-day-old broiler chickens in this study was 20 times the concentration that was recommended to be administered to day-old chicks. The level of challenge used in this study was based on levels we have previously used [19,20] The vaccine contained live *Eimeria* oocysts of *E. acervulina, E. maxima, E. maxima* MFP, *E. mivati,* and *E. tenella*.

### 2.3. Ileal Digesta and Excreta Collection

After euthanasia, ileal digesta from the distal two-thirds of the ileum was gently flushed with distilled water into a clean pre-labeled plastic container on days 21 and 28 (4 birds/cage). Excreta samples were collected on days 19, 20, and 21 and 26, 27, 28 using the total collection method, as previously described [17]. Excreta samples were dried in a forced-air oven at 55 °C for six days. Dried excreta samples from days 19 to 21 of collection were pooled per cage for day 21 chemical analysis, while dried excreta samples collected on days 26 to 28 were also pooled for day 28 chemical analysis. Dried excreta samples and diets were ground to pass through a 0.5 mm screen using a mill grinder (Wiley Mill Standard Model No. 3, Arthur H. Thomas Co., Philadelphia, PA, USA). Ileal digesta were freeze-dried and ground using a coffee grinder.

### 2.4. Histological Analysis

About an inch of the mid-section of the duodenum and the jejunum was taken on day 21 from one bird per cage (closest to the average weight of the four birds selected for sampling) to determine the morphometric changes associated with the different treatments, especially CVC. The CVC used in this study is capable of affecting the entire gastrointestinal tract. Treatment effects on the histology of the jejunum have been presented in [17,18]. Samples were processed for histology at the University of Kentucky’s Animal Diagnostics Lab (ADL). The villi height and crypt depth of ten distinct villi were measured per slide at 10x (upright clinical microscope, Model Eclipse Ci-E, Nikon Corporation, Tokyo, Japan). In addition to the villi height (VH), the villi width (VW) and crypt depth (CD) were also determined. The villi height to crypt depth ratio (VH:CD) was then calculated from the measured values.

### 2.5. Chemical Analysis

The titanium, dry matter (DM), gross energy (GE), nitrogen (N), calcium (Ca), and phosphorus (P) in the diets and the ileal digesta samples were determined, while the DM, Ca, and P in the excreta samples were also determined in duplicates. The DM (method 934.01) of the diets, the ileal digesta, and the excreta were determined by drying the samples at 110 °C for 16 h [21]. Samples’ N (method 990.03) contents were determined using the combustion method (model FP2000, Leco Corp., St. Joseph, MI, USA) [22], with EDTA as the internal standard. The GE values of the different samples were determined using a bomb calorimeter (Parr adiabatic bomb calorimeter, model 6200, parr instruments, Moline, IL, USA) using benzoic acid as the calibration standard. Calcium concentrations in the samples were determined using the flame atomic absorption spectroscopy method (Varian Spectr. AA 220FS, Varian Australia Pty Ltd., Mulgrave, Australia), while the titanium concentrations in the diets, the ileal digesta, and the excreta samples were determined as described by [23].

### 2.6. Calculations and Statistical Analysis

The apparent ileal energy and nutrient digestibility (AID) and apparent Ca and P utilization (ANU) were calculated using the following equation [24]:

$$\mathrm{AID}\;\mathrm{of}\;\mathrm{ANU},\left(\%\right)=[1-\left(\frac{TiI}{TiO}\right)\times \left(\frac{No}{Ni}\right)]100$$

where TiI is the titanium concentration (in %) in the diets while TiO is the titanium concentration in the ileal digesta (for AID determination) or the excreta (for ANU determination). No is the concentrations (in %) of nutrient or energy in the ileal digesta or the excreta (only Ca and P) while Ni is the concentrations (in %) of nutrient or energy in the diets. The digestible energy (DE) was calculated as the product of energy digestibility (%) and the gross energy of the respective diets.

Data were statistically analyzed using the GLM procedure of SAS (SAS Inst. Inc. Cary, NC, USA, 2006). In order to take into consideration the nature of the respective diet types (isocaloric and isonitrogenous), data were analyzed using codes that were appropriate for a 2 (CVC, yes vs. no) × 2 (enzyme supplementation, yes or no) factorial within each of the two diet types (CS-based vs. WCS-based diets). The cage served as the experimental unit, except for histology data, where one bird per cage was the experimental unit. The number of replicates was seven per treatment, unless otherwise stated. Outliers (data outside of mean ± 3 SD) were removed from the data prior to statistical analysis and, where necessary, mean separation was performed using Tukey’s test; the level of significance was set at *p* < 0.05. Irrespective of whether or not the interaction was significant, the main and simple effect data are presented.

## 3. Results

The activity levels of the exogenous enzymes used in this study were determined prior to the start of the feeding trial (DSM Nutritional Products, Parsippany NJ, USA). The levels of glucanase in the control diets were 3.2 (CS-based diet) and 5.0 (WCS-based diets) FBG/kg. For the exogenous-enzyme-supplemented diets, the measured activity levels of glucanase from Ronozyme^®^ VP supplemented CS- and WCS-based diets were 18.4 and 24.5 FBG/kg, respectively. The corresponding activity levels of xylanase from Ronozyme^®^ WX2 supplementation were 259 and 311 FXU/kg for the CS- and WCS-based diets, respectively [17,18]. The respective levels of xylanase in the non-supplemented control diets were below the detection limit (Table 1).

### 3.1. Performance

The performance of birds fed corn–SBM-based diets is reported in Table 2. During the first 7 days post-CVC (day 14–21), CVC decreased (*p* < 0.05) the average BWG (19.7%), feed intake (7.9%), and feed efficiency (12.8%), while birds on the enzyme-supplemented diet had higher (*p* < 0.05) average BWG (5.3%; day 14–21) compared to birds fed diets without enzyme supplementation. Fourteen days post-CVC, enzyme supplementation resulted in higher (*p* < 0.05) average BWG and feed intake compared with CVC birds on diets without enzyme supplementation (Table 2). The CVC birds fed WCS-based diets had lower (*p* < 0.05) average BWG, feed intake, and feed efficiency 7 days post-CVC compared with the unchallenged birds fed diets without enzyme supplementation (Table 3). However, the performance of birds fed the WCS-based diets showed that CVC increased (*p* < 0.05) the average BWG and feed efficiency 14 days post-CVC (Table 3).

### 3.2. Nutrient and Energy Digestibility and Utilization

The apparent ileal digestibility of DM, N, energy, and DE 7 days post-CVC was lower (*p* < 0.05) irrespective of diet type (Table 4). In birds fed the CS-based diets, CVC decreased (*p* < 0.05) the DE, DM, and energy digestibility 14 d post-CVC, while both the CVC and enzyme supplementation resulted in higher (*p* < 0.05) ileal Ca digestibility (Table 5). For birds on the WCS-based diets, CVC decreased (*p* < 0.05) the apparent ileal DE, DM, N, and energy digestibility but increased (*p* < 0.05) the apparent ileal Ca digestibility by 16% 14 d post-CVC (Table 5).

Apparent Ca and P utilization decreased (*p* < 0.05) with CVC 7 d post-CV challenge, while it decreased (*p* < 0.05) the apparent Ca (17.6%) and increased (*p* < 0.05) the apparent P (4.2%) utilization in birds fed the CS-based diets 14 days post-challenge (Table 6). Interactions between CVC and enzyme supplementation showed that enzyme supplementation increased (*p* < 0.05) Ca and P utilization in the unchallenged birds fed the WCS-based diets 7 days post-CVC, while enzyme supplementation increased (*p* < 0.05) P utilization in the unchallenged birds fed the WCS-based diets. Enzyme supplementation increased (*p* < 0.05) Ca and P utilization in the CVC birds fed WSC-based diets 14 days post-CVC (Table 6).

### 3.3. Histology

Seven days post-CVC, the duodenal VH and VH:CD were decreased (*p* < 0.05) by the CVC, and the CD was increased (*p* < 0.05) in birds fed the CS-based diets, while enzyme supplementation reduced (*p* < 0.05) the VW in birds fed CS-based diets (Table 7). In birds fed the WCS-based diets, the duodenal VH and VH:CD were decreased (*p* < 0.05) by the CVC, while CD was increased (*p* < 0.05). Enzyme supplementation influenced the CD (increased; *p* < 0.05) and VH:CD (decreased; *p* < 0.05) (Table 7).

In the jejunum of birds fed the CS-based diets (Table 8), CVC increased (*p* < 0.05) the VW relative to that of the unchallenged birds; however, the exogenous enzyme reduced (*p* < 0.05) the VW of the CVC birds compared to the CVC birds on diets without enzyme supplementation. The CD increased (*p* < 0.05) while the VH:CD decreased (*p* < 0.05) in the CVC birds fed the CS-based diets (Table 8).

For birds fed the WCS-based diets, the VW decreased (*p* < 0.05) with the CVC, while the interactions between the CVC and exogenous enzyme supplementation resulted in a higher (*p* < 0.05) CD compared with the unchallenged birds, irrespective of exogenous enzyme supplementation (Table 8). Challenged birds fed the enzyme-supplemented diet had a lower (*p* < 0.05) VH:CD compared with the unchallenged birds (Table 8).

## 4. Discussion

With the increasing growth of the human population comes the challenge of food insecurity associated with increased demand for animal protein. In order to address this challenge, more animal protein has to be produced while decreasing arable land for producing plant-based feed ingredients, which constitutes more than 90% of poultry feed. In addition to this, poultry faces the challenge of not being able to adequately handle the anti-nutritional factors, such as phytic acid and NSP, found in plant-based feed ingredients. The annual financial loss to the poultry industry because of parasitic protozoan *Eimeria* infection has been reported to run into billions of dollars [2,25], and the destruction it causes to the GIT of birds allows other opportunistic bacteria to cause further infection of the tract [26,27]. The presence of these anti-nutrients in the poultry diet not only reduces the birds’ ability to efficiently utilize nutrients and energy in the diet but also affords bacteria in the hindgut access to relatively higher levels of undigested nutrients and energy, and this could further negatively impact the health of the bird’s GIT. Supplementation with exogenous enzymes could assist in reducing the potentially negative effects of high levels of energy and nutrients reaching the hindgut [8,28], thereby minimizing the negative effects of bacteria on the GIT.

The BWG of birds fed the enzyme-supplemented CS-based diet increased by 5.3% (days 14–21), which means that despite the fact that the diet that was fed met or exceeded nutrient and energy requirements, the exogenous enzyme was able to further improve performance. The lack of a significant effect of enzyme supplementation in most of the other performance response variables measured in this study could be attributed to the fact that the diet’s nutrient level was adequate for birds of this age. The negative effect of the *Eimeria* sp. challenge on BWG, feed intake, and nutrient digestibility and utilization has been previously documented [13,17,19,29]. The decrease in feed efficiency 7 days post-CVC is an indication of the challenges that the birds faced with regards to nutrient digestion and absorption as a result of the negative effects of the protozoan parasites on the intestinal wall, including the destruction of the villi that are responsible for nutrient absorption. Interestingly, during the recovery phase (14 days post-challenge), BWG (7.5%) and feed efficiency (5.3%) improved in the CVC-challenged birds fed WCS-based diets. This compensation during the recovery phase has been previously reported and can be attributed to the fact that because the birds were raised in cages, the ability of the protozoan oocysts to be recycled through the excreta or littler was not available to the birds.

For the CS-based diets, interactions between CVC and enzyme supplementation showed that the average BWG 14 days post-CVC was not different from that of the unchallenged birds with or without enzyme supplementation, but it was higher than in CVC birds fed the diet without enzyme supplementation by about 5.1%. This means the reduction in the average BWG as a result of CVC was quickly restored with enzyme supplementation during the recovery phase. This was achieved with significantly more feed intake in the CVC birds fed enzyme-supplemented diets. One interesting observation from the performance data was that unlike in the CS-based diets where the main effect of CVC was not significant on the average BWG and feed efficiency 14 days post-CVC, CVC birds fed the WCS-based diets had higher average BWG (7.5%) and feed efficiency (5.3%) than the unchallenged birds 14 days post-challenge. This could be due to the fact that the inclusion of wheat in their diet resulted in relatively higher undigested and unabsorbed energy and nutrients from the diet as a result of higher digesta viscosity [17] reaching the hindgut during the peak period of infection, which increases the potential for opportunistic bacterial proliferation [26,27]. However, during the recovery phase, digestion and absorption of nutrients and energy in the diet increased as a result of the break in the life cycle of the protozoan (the inability of the bird to recycle oocysts because of lack of access to the excreta). This would result in less nutrients reaching the hindgut. The jejunal digesta viscosity from this study, as reported earlier [17], showed that 7 days post-CVC, the viscosity of the contents of the jejunum from the WCS-based diet was 14% higher compared to that from birds fed the CS-based diet (2.846 vs. 2.496 cP). This value dropped to 10.0% 14 days post-CVC, with about 29.6% reduction in digesta viscosity leading to higher access of digestive enzymes to the digesta and hence higher nutrient digestion. However, 14 days post-CVC, these birds were able to compensate for the loss in average BWG and feed efficiency after the effect of the *Eimeria* sp. had considerably decreased, leading to relatively healthy GIT and villi as well as improved digestion and absorption of nutrients and energy, with fewer nutrients for opportunistic bacteria to thrive on [26,30,31]. Another possibility is the potential effect of wheat playing some role in the type and composition of the microbiome in the hindgut, with a potential for some of the NSP components in wheat to have some prebiotic effect in the hindgut.

Two of the four *Eimeria sp*. in the CVC used in this study were *Eimeria maxima* (jejunum and ileum) and *Eimeria acervulina* (duodenum and jejunum), both of which infect the midgut [32]. The effect of these species is expected to be pronounced on the digestion and absorption of energy and nutrients from the diets in the midgut [20,33]. Ileal digestibility values of DM, energy, and DE were reduced in the CVC birds (7 and 14 days post-challenge) irrespective of the diet type. This observation is similar to what has been previously reported [19,34] in broiler chickens challenged with *Eimeria* sp. For N digestibility, a significant reduction was observed in the CVC birds irrespective of the diet and stage of infection, except for birds fed the CS-based diets, where there was no difference in N digestibility between the challenged and the control birds 14 days post-challenge. This could be an indication of an increase in amino acid (N) absorption during the recovery phase compared to challenged birds fed the WCS-based diets. The decrease in ileal energy and nutrient digestibility in the CVC birds could be attributed to several factors, including the destruction of the intestinal epithelia along the GIT, the thickening of the intestinal mucosa, the shortening of the villi, which ultimately would lead to a reduction in the available area for interaction between the digesta and the absorptive surface of the villi, but, more importantly, the development and repair of the destroyed structure of the intestinal tissue, as seen by the increase in crypt depth, which becomes a priority [35,36]. It is worth noting that the effect of the CVC on VH was more pronounced in the duodenum compared to the jejunum, which may indicate that the mix of *Eimeria* sp. used in this study may have exerted a more negative effect on nutrient and energy digestion in the duodenum. Aside from the reduction in the villi height and the VH:CD, the negative effect of the changes in the intestinal dynamics, including changes in the digesta passage rate, which reduced the activities of the brush border membrane enzyme, as well as the leaky tight junction, may explain this reduction in digestibility.

On the contrary, during the peak of infection from CVC, ileal Ca and P digestibility values were significantly reduced by CVC irrespective of the diet type. However, 14 days post-challenge, apparent ileal P digestibility increased by 4.2% (CS-based diet). The interaction between the two main factors in birds fed the WCS-based diet revealed that enzyme supplementation of the diets fed to the CVC birds significantly ameliorated the negative effect of CVC on Ca (13.8%) and P (9.8%) digestibility in birds fed the WCS-based diet. This observation could have been brought about by several factors, including the potential ability of both the carbohydrase and phytase enzymes to have a synergistic effect on Ca and P digestibility during the recovery phase, as well as the nature of the diet, with digesta from the wheat-based diet being relatively high in viscosity, making the carbohydrase enzymes more effective compared to the CS-based diet. Furthermore, the high level and activities of Ca binding protein in the small intestine, changes in the active vitamin D dynamics, as well as an increase in demand for Ca during the recovery phase may have had an impact. Other reports [19,29] showed either an increase or a tendency for Eimeria infection to increase Ca digestibility.

## 5. Conclusions

Results from this study confirmed that for birds raised in cages, the most detrimental effect of *Eimeria* infection occurs within the first week, typically between 5 and 7 days post-challenge, and this is obvious in the performance and nutrient digestibility data. However, due to the birds’ inability to recycle oocysts because they were raised in cages, the significant negative effects of CVC that were observed 7 days post-challenge were muted by 14 days post-CVC. An interesting result from this study was the fact that the rebound in BWG and feed efficiency during the recovery phase differed, with challenged birds fed the WCS-based diets having significantly higher BWG and feed efficiency despite consuming identical amounts of feed as the unchallenged birds. The implication of this is important for poultry producers located in areas with potentially high incidence of coccidiosis. Another important finding from this study was that the only interaction between the two main factors (CVC and enzyme supplementation) was observed for Ca and P utilization, and these interactions were confined to the WCS-based diets 7 and 14 days post-CVC. On day 7 post-challenge, enzyme supplementation significantly increased Ca and P utilization in the unchallenged birds, while 14 days post-challenge, enzyme supplementation resulted in significant increases in Ca and P utilization in CVC birds. These interactions may have contributed to the improved performance observed in birds fed the WCS-based diets 14 days post-challenge. Finally, data from these studies showed that CVC birds had the ability to significantly increase P utilization 14 days post-challenge compared to Ca utilization, which still lagged behind that of the unchallenged birds 14 days post-CVC.

## Figures and Tables

**Table 1:** Feed ingredient composition and analyzed nutrient contents of the experimental diets ^1^.

Ingredients, g/kg (as-fed)	Without Enzymes		With Enzymes	
	CS	WCS	CS	WCS
Corn	639.6	438.6	619.6	418.6
Soybean meal, 48% CP	285.0	195.5	285.0	195.5
Wheat (hard red)	0.0	300.0	0.0	300.0
Soy oil	30.0	20.5	30.0	20.5
Others ^2^	40.4	40.4	40.4	40.4
Titanium dioxide	5.0	5.0	5.0	5.0
Ronozyme^®^ WX2 premix ^3^	0.0	0.0	10.0	10.0
Ronozyme^®^ VP premix ^4^	0.0	0.0	10.0	10.0
Total	1000	1000	1000	1000
Analyzed nutrient and energy composition ^5^				
Gross energy, kcal/kg	4016	3918		
Dry matter, g/kg	895.0	889.5		
Crude protein (nitrogen × 6.25), g/kg	197.2	174.7		
Calcium, g/kg	9.4	9.2		
Phosphorus, g/kg	7.1	6.9		
Analyzed enzyme activities ^6^				
Glucanase, FBG/kg	3.2	5.0	18.4	24.5
Xylanase, FXU/kg	ND ^7^	ND	259	311

^1^ CS = corn–SBM; WCS = wheat–corn–SBM. ^2^ Others (40.4 g/kg) include dicalcium phosphate (17.6 g/kg), limestone, 10.5 g/kg), vitamin–mineral premix (2.5 g/kg), salt (NaCl; 4.1 g/kg), DL-methionine (3.0 g/kg), L-lysine HCL (2.0 g/kg), and L-threonine (5.0 g/kg). Details of the diets are referenced in [17,18]. The vitamin–mineral premix supplied the following at 2.5 g per kilogram of the diet: 11 025 IU of vitamin A, 3 528 IU of vitamin D, 33 IU of vitamin E, 0.91 mg of vitamin K, 2.21 mg of thiamin, 7.72 mg of riboflavin, 55 mg of niacin, 18 mg of pantothenate, 5 mg of vitamin B-6, 0.22 mg of d-biotin, 1.10 mg of folic acid, 478 mg of choline, 0.03 of vitamin B-12, 75 mg of Zn, 40 mg of Fe, 64 mg of Mn, 10 mg of Cu, 1.85 mg of I, and 0.30 mg of Se. ^3^ The premix (ground-corn-based) supplied 0.1 g of Ronozyme^®^ WX2/kg of the diet. The enzyme was added at the expense of ground corn. ^4^ The premix (ground-corn-based) supplied 0.25 g of Ronozyme^®^ VP/kg of the diet. The enzyme was added at the expense of ground corn. ^5^ The diets with enzyme supplementation were produced from the same basal diet as diets without enzyme supplementation. ^6^ Analysis was conducted at the DSM lab in NJ, USA. ^7^ Below detection limit.

**Table 2:** Main and simple effects of coccidia vaccine challenge (CVC) and exogenous enzyme supplementation on performance of broiler chickens fed corn-soybean meal-based diets 7 (d 21) and 14 (d 28) days post-challenge ^1^.

CVC	Enzyme	Body Weight, g/bird			Body Weight Gain, g/bird		Feed Intake, g/bird		Feed Efficiency, g/kg	
		D 14	D 21	D 28	Day 14–21	Day 21–28	Day 14–21	Day 21–28	Day 14–21	Day 21–28
		Means for Main Effect of CVC								
−		393	820 ^a^	1508 ^a^	426 ^a^	677	582 ^a^	933	732 ^a^	726
+		394	736 ^b^	1398 ^b^	342 ^b^	666	536 ^b^	916	638 ^b^	728
		Means for Main Effect of Enzyme								
	−	395	769	1441	374 ^b^	669	555	916	685	731
	+	392	787	1465	394 ^a^	675	564	933	686	723
		CVC × Enzyme								
−	−	394	818	1518 ^a^	424	688 ^a^	588 ^a^	943 ^a^	722	729
+	−	396	720	1364 ^b^	324	650 ^b^	522 ^c^	889 ^b^	648	732
−	+	392	822	1497 ^a^	428	667 ^ab^	577 ^ab^	923 ^a^	743	723
+	+	392	752	1433 ^b^	360	683 ^a^	550 ^bc^	943 ^a^	629	724
	SD ^2^	18.42	27.00	52.17	24.82	33.38	22.54	45.46	33.68	19.58
		Probability								
	CVC	0.911	<0.001	<0.001	<0.001	0.395	<0.001	0.330	<0.001	0.804
	Enzyme	0.693	0.088	0.241	0.042	0.627	0.301	0.338	0.934	0.344
	CVC × Enzyme	0.879	0.192	0.031	0.101	0.043	0.029	0.039	0.131	0.954

^a–c^ Means within the same column with different superscripts are different. ^1^ Number of replicates for the simple effect of −CVC × −Enzyme = 7, +CVC × −Enzyme = 7, −CVC × +Enzyme = 7, +CVC × +Enzyme = 7. ^2^ Standard deviation.

**Table 3:** Main effects of coccidia vaccine challenge (CVC) and exogenous enzyme supplementation on performance of broiler chickens fed wheat–corn–soybean meal-based diets 7 (d 21) and 14 (d 28) days post-challenge ^1^.

CVC	Enzyme	Body Weight, g/bird			Body Weight Gain, g/bird		Feed Intake, g/bird		Feed Efficiency, g/kg	
		D 14	D 21	D 28	Day 14–21	Day 21–28	Day 14–21	Day 21–28	Day 14–21	Day 21–28
		Means for Main Effect of CVC								
−		393	788 ^a^	1390 ^a^	396 ^a^	599 ^b^	613 ^a^	933	647 ^a^	642 ^b^
+		392	706 ^b^	1346 ^b^	313 ^b^	644 ^a^	567 ^b^	952	553 ^b^	676 ^a^
		Means for Main Effect of Enzyme								
	−	392	746	1374	352	628	592	947	593	662
	+	392	748	1362	357	615	587	938	606	656
	SD ^2^	15.69	23.89	39.91	15.97	28.23	18.86	42.12	15.75	14.72
		Probability								
	CVC	0.896	<0.001	0.008	<0.001	<0.001	<0.001	0.238	<0.001	<0.001
	Enzyme	0.918	0.789	0.461	0.409	0.244	0.555	0.568	0.056	0.242
	CVC × Enzyme	0.790	0.313	0.440	0.354	0.706	0.058	0.742	0.399	0.926

^a,b^ Means within the same column with different superscripts are different. ^1^ Number of replicates was 14 each except for +CVC (13) and −Enzyme (13). ^2^ Standard deviation.

**Table 4:** Main effects of coccidia vaccine challenge (CVC) and exogenous enzyme supplementation on apparent ileal dry matter (DM), nitrogen (N), calcium (Ca), phosphorus (P), energy digestibility, and apparent ileal digestible energy in 21-day-old broiler chickens fed corn-soybean meal- and wheat–corn-soybean meal-based diets (7 days post-challenge).

CVC	Enzyme	Corn–Soybean-Meal-Based Diet ^1^						Wheat–Corn–Soybean-Meal-Based Diet ^2^					
		DM, %	N, %	Ca, %	P, %	Energy, %	DE, kcal/kg	DM, %	N, %	Ca, %	P, %	Energy, %	DE, kcal/kg
		Means for Main Effect of CVC						Means for Main Effect of CVC					
−		71.1 ^a^	80.9 ^a^	38.0	47.4	76.1 ^a^	3820 ^a^	71.0 ^a^	81.0 ^a^	37.8	46.0	75.9 ^a^	3761 ^a^
+		51.1 ^b^	66.5 ^b^	40.9	44.9	55.3 ^b^	2777 ^b^	51.9 ^b^	68.3 ^b^	41.5	42.8	56.5 ^b^	2801 ^b^
		Means for Main Effect of Enzyme						Means for Main Effect of Enzyme					
	−	60.9	72.9	37.9	45.0	65.4	3283	61.9	74.3	39.4	44.2	66.5	3294
	+	61.4	74.5	41.1	47.3	66.0	3313	61.0	75.0	39.8	44.6	66.0	3268
	SD ^3^	3.51	2.97	7.05	4.56	3.24	162.0	4.96	3.44	5.60	4.66	4.65	230.3
		Probability						Probability					
	CVC	<0.001	<0.001	0.319	0.179	<0.001	<0.001	<0.001	<0.001	0.109	0.091	<0.001	<0.001
	Enzyme	0.763	0.208	0.270	0.231	0.654	0.645	0.675	0.617	0.864	0.841	0.785	0.785
CVC × Enzyme		0.647	0.350	0.939	0.556	0.608	0.601	0.234	0.481	0.649	0.258	0.316	0.315

^a,b^ Means within the same column with different superscripts are different. ^1^ Number of replicates was 14 each except for −CVC (13), +CVC (12); −Enzyme (12), and +Enzyme (13). ^2^ Number of replicates was 14 each except for −CVC (13), +CVC (12); −Enzyme (12), and +Enzyme (13). ^3^ Standard deviation.

**Table 5:** Main effects of coccidia vaccine challenge (CVC) and exogenous enzyme supplementation on apparent ileal dry matter (DM), nitrogen (N), calcium (Ca), phosphorus (P), energy digestibility, and apparent ileal digestible energy in 28-day-old broiler chickens fed corn-soybean meal- and wheat–corn–soybean meal-based diets (14 days post-challenge).

CVC	Enzyme	Corn–Soybean-Meal-Based Diet ^1^						Wheat–Corn–Soybean-Meal-Based Diet ^2^					
		DM, %	N, %	Ca, %	P, %	Energy, %	DE, kcal.kg	DM, %	N, %	Ca, %	P, %	Energy, %	DE, kcal/kg
		Means for Main Effect of CVC						Means for Main Effect of CVC					
−		77.8 ^a^	86.4	36.4 ^b^	51.5	82.3 ^a^	4133 ^a^	78.5 ^a^	87.6 ^a^	38.8 ^b^	53.4	82.9 ^a^	4108
+		75.8 ^b^	85.3	46.5 ^a^	53.1	80.4 ^b^	4034 ^b^	77.4 ^b^	85.9 ^b^	45.0 ^a^	51.5	82.0 ^b^	4060
		Means for Main Effect of Enzyme						Means for Main Effect of Enzyme					
	−	77.0	86.2	38.5 ^b^	51.9	81.6	4096	77.9	86.6	39.9	52.2	82.4	4084
	+	76.5	85.5	44.4 ^a^	52.7	81.1	4071	78.4	86.8	44.0	52.7	82.5	4084
	SD ^3^	1.23	1.57	5.89	2.86	1.10	55.31	1.15	0.80	7.21	3.41	1.23	60.96
		Probability						Probability					
	CVC	<0.001	0.093	<0.001	0.166	<0.001	<0.001	0.005	<0.001	0.035	0.165	0.049	0.050
	Enzyme	0.283	0.303	0.013	0.472	0.253	0.243	0.274	0.531	0.150	0.730	0.958	0.976
CVC × Enzyme		0.620	0.124	0.114	0.086	0.945	0.942	0.229	0.265	0.170	0.321	0.387	0.375

^a,b^ Means within the same column with different superscripts are different. ^1^ Number of replicates was 14 each except for DM where −CVC and +CVC = 13 each and −Enzyme = 12. For energy, the number of replicates for −CVC, −Enzyme, and +Enzyme = 13 each. For P, the number of replicate for −CVC and −Enzyme was 13 each, while for DE, it was 13 each for −CVC and −Enzyme. For N, +CVC and −Enzyme = 13. ^2^ Number of replicates was 14 each except for N where −CVC, +CVC, −Enzyme, and +Enzyme = 12 each; DM, where −CVC, +CVC, −Enzyme, and +Enzyme = 13 each; energy and DE, where +CVC and −Enzyme = 13 each; and for Ca, where +CVC and +Enzyme = 13 each. ^3^ Standard deviation.

**Table 6:** Main and simple effects of coccidia vaccine challenge (CVC) and exogenous enzyme supplementation on apparent calcium (Ca) and phosphorus (P) utilization in 21-day-old (7 days post-challenge) and 28-day-old (14 days post-challenge) broiler chickens fed corn-soybean (CS) meal- and wheat–corn–soybean (WCS) meal-based diets.

CVC	Enzyme	Day 21 (7 Days Post-Challenge)				Day 28 (14 Days Post-Challenge)			
		CS-Based Diet ^1^		WCS-Based Diet ^2^		CS-Based Diet ^3^		WCS-Based Diet ^4^	
		Ca, %	P, %	Ca, %	P, %	Ca, %	P, %	Ca, %	P, %
		Means for Main Effect of CVC		Means for Main Effect of CVC		Means for Main Effect of CVC		Means for Main Effect of CVC	
−		50.8 ^a^	52.0 ^a^	49.6 ^a^	46.4 ^a^	40.8 ^a^	44.8 ^b^	39.4 ^a^	40.4 ^b^
+		40.8 ^b^	44.4 ^b^	36.5 ^b^	40.0 ^b^	33.6 ^b^	46.7 ^a^	35.6 ^b^	44.8 ^a^
		Means for Main Effect of Enzyme		Means for Main Effect of Enzyme		Means for Main Effect of Enzyme		Means for Main Effect of Enzyme	
	−	46.5	48.1	42.1	42.6 ^b^	37.2	46.0	36.4	41.3 ^b^
	+	45.0	48.3	43.9	43.8 ^a^	36.5	45.4	38.6	43.9 ^a^
		CVC × Enzyme				CVC × Enzyme		CVC × Enzyme	
−	−	51.7	52.0	47.1 ^a^	44.7 ^b^	41.8	45.5	39.5 ^a^	40.0 ^b^
+	−	41.4	44.1	37.1 ^b^	40.5 ^c^	32.5	46.6	33.3 ^b^	42.7 ^b^
-	+	49.8	52.0	52.1 ^a^	48.1 ^a^	38.5	44.0	39.3 ^a^	40.9 ^b^
+	+	40.2	44.7	35.8 ^b^	39.6 ^c^	34.6	46.9	37.9 ^a^	46.9 ^a^
	SD ^5^	3.43	2.00	2.96	1.36	3.84	2.37	2.79	1.94
		Probability		Probability		Probability		Probability	
	CVC	<0.001	<0.001	0.001	<0.001	<0.001	0.047	0.007	<0.001
	Enzyme	0.273	0.747	0.157	0.037	0.663	0.523	0.062	0.003
CVC × Enzyme		0.837	0.747	0.020	0.001	0.091	0.350	0.042	0.040

^a–c^ Means within the same column with different superscripts are different. ^1^ Number of replicates for simple effect of −CVC × −Enzyme = 6, +CVC × −Enzyme = 7, −CVC × +Enzyme = 6, +CVC × +Enzyme = 6. ^2^ n for simple effect of −CVC × −Enzyme = 5, +CVC × −Enzyme = 5, −CVC × +Enzyme = 7, +CVC × +Enzyme = 6. ^3^ n for simple effect of −CVC × −Enzyme = 6, +CVC x +Enzyme = 7, −CVC × +Enzyme = 6, +CVC × +Enzyme = 7. ^4^ Number of replicates for simple effect of −CVC × −Enzyme = 6, +CVC × −Enzyme = 6, −CVC × +Enzyme = 7, +CVC × +Enzyme = 7. ^5^ Standard deviation.

**Table 7:** Main effects of coccidia vaccine challenge (CVC) and exogenous enzyme supplementation on duodenal villi height (VH), villus width (VW), crypt depth (CD), and villi height-to-crypt-depth ratio (VH:CD) in 21-day-old broiler chickens fed corn–soybean meal- (CS) or wheat–corn–soybean meal- (WCS) based diets (7 days post-challenge).

CVC	Enzyme	Corn–Soybean-Meal-Based Diet ^1^				Wheat–Corn–Soybean-Meal-Based Diet ^2^			
		VH, µm	VW, µm	CD, µm	VH:CD	VH, µm	VW, µm	CD, µm	VH:CD
		Means for Main Effect of CVC				Means for Main Effect of CVC			
−		2929 ^a^	310	280 ^b^	10.78 ^a^	3017 ^a^	305	250 ^b^	12.25 ^a^
+		2449 ^b^	302	434 ^a^	5.67 ^b^	2532 ^b^	293	418 ^a^	6.11 ^b^
		Means for Main Effect of Enzyme				Means for Main Effect of Enzyme			
	-	2650	323 ^a^	368	8.00	2896	298	313 ^b^	10.20 ^a^
	+	2728	289 ^b^	346	8.46	2653	300	355 ^a^	8.16 ^b^
	SD ^3^	2.01.7	31.5	46.6	1.317	3.05.0	36.3	35.1	1.167
		Probability				Probability			
	CVC	<0.0001	0.519	<0.0001	<0.0001	0.001	0.430	<0.0001	<0.0001
	Enzyme	0.345	0.014	0.247	0.386	0.074	0.885	0.009	0.001
CVC × Enzyme		0.852	0.738	0.575	0.802	0.220	0.102	0.432	0.050

^a,b^ Means within the same column with different superscripts are different. ^1^ Number of replicates for −CVC and +Enzyme was 13 each while it was 12 each for +CVC and −Enzyme. ^2^ Number of replicates was 11 for −CVC, 12 for +CVC, 10 for −Enzyme, and 13 for +Enzyme. ^3^ Standard deviation.

**Table 8:** Main and simple effects of coccidia vaccine challenge (CVC) and exogenous enzyme supplementation on jejunal villi height (VH), villus width (VW), crypt depth (CD), and villi height-to-crypt-depth ratio (VH:CD) in 21-day-old broiler chickens fed corn–soybean meal- (CS) or wheat–corn–soybean meal- (WCS) based diets (7 days post-challenge).

CV	Enzyme	Corn–Soybean-Meal-Based Diet ^1^				Wheat–Corn–Soybean-Meal-Based Diet ^2^			
		VH, µm	VW, µm	CD, µm	VH:CD	VH, µm	VW, µm	CD, µm	VH:CD
		Means for Main Effect of CVC				Means for Main Effect of CVC			
-		1532	152 ^b^	180 ^b^	8.41 ^a^	1601	164 ^b^	179 ^b^	9.01 ^a^
+		1550	234 ^a^	241 ^a^	6.65 ^b^	1460	211 ^a^	242 ^a^	6.04 ^b^
		Means for Main Effect of Enzyme				Means for Main Effect of Enzyme			
	-	1571	221 ^a^	216	7.52	1544	194	209	7.59
	+	1511	166 ^b^	205	7.53	1517	181	212	7.45
		CVC × Enzyme				CVC × Enzyme			
-	-	1600	157 ^c^	175	9.30	1629	173	192 ^bc^	8.50 ^ab^
+	-	1543	285 ^a^	256	5.75	1459	216	226 ^ab^	6.68 ^bc^
-	+	1464	148 ^c^	184	7.51	1573	155	166 ^c^	9.51 ^a^
+	+	1558	184 ^b^	226	7.56	1460	207	258 ^a^	5.40 ^c^
	SD ^3^	314.4	22.6	32.0	1.285	260.8	21.8	35.8	1.316
		Probability				Probability			
	CVC	0.877	<0.001	<0.001	0.002	0.182	<0.001	<0.001	<0.001
	Enzyme	0.619	<0.001	0.403	0.403	0.790	0.145	0.840	0.795
Enzyme × CVC		0.532	<0.001	0.129	0.129	0.781	0.619	0.048	0.038

^a–c^ Means within the same column with different superscripts are different. ^1^ Number of replicates for simple effect of −CVC × −Enzyme = 7, +CVC × −Enzyme = 6, −CVC × +Enzyme = 6, +CVC × +Enzyme = 7. ^2^ Number of replicates for simple effect of −CVC × −Enzyme = 7, +CVC × −Enzyme = 6, −CVC × +Enzyme = 6, +CVC × +Enzyme = 7. ^3^ Standard deviation.

## Data Availability

The data presented in this study are available upon request from the corresponding author.
